# Discordance across Phenotypic and Molecular Methods for Drug Susceptibility Testing of Drug-Resistant *Mycobacterium tuberculosis* Isolates in a Low TB Incidence Country

**DOI:** 10.1371/journal.pone.0153563

**Published:** 2016-04-20

**Authors:** Suhail Ahmad, Eiman Mokaddas, Noura Al-Mutairi, Hanaa S. Eldeen, Shirin Mohammadi

**Affiliations:** 1 Department of Microbiology, Faculty of Medicine, Kuwait University, Safat, Kuwait; 2 Kuwait National TB Reference Laboratory, Shuwaikh, Kuwait; University of Padova, Medical School, ITALY

## Abstract

With increasing incidence of multidrug-resistant tuberculosis (MDR-TB), accurate drug susceptibility testing (DST) of *Mycobacterium tuberculosis* to first-line drugs has become crucial for proper patient management. We evaluated concordance of DST results for 70 *M*. *tuberculosis* isolates across two phenotypic and two molecular methods: BACTEC 460TB, MGIT 960 system, GenoType MTBDR*plus* and DNA sequencing of gene segments most commonly implicated in conferring resistance to anti-TB drugs. Most (84%) *M*. *tuberculosis* isolates were multidrug-resistant. Twenty-four isolates yielded discrepant DST results. For rifampicin, isoniazid and streptomycin, 96%, 97% and 93% of isolates, respectively, were susceptible or resistant by all four methods, whereas for ethambutol, this agreement was observed for only 76% of isolates (*P* <0.05 for rifampicin or isoniazid or streptomycin versus ethambutol). Occurrence of rare mutations in three isolates that confer low-level resistance caused lower agreement for rifampicin among the four methods (kappa coefficient (κ) range, 0.84 to 0.95). For isoniazid, there was perfect agreement among phenotypic methods and molecular methods (κ, 1.00) but lower agreement between phenotypic and molecular methods. Three isolates were detected as polydrug-resistant by MGIT 960 system but as multidrug-resistant by DNA sequence-based method. The agreement was higher for streptomycin among the two phenotypic methods (κ, 0.97) while targeted sequencing yielded lower agreement (κ range, 0.86 to 0.89). The discrepancy for ethambutol resulted largely due to lower concordance of MGIT 960 results (κ range, 0.53 to 0.64). The MGIT 960 system is an accurate method for DST of *M*. *tuberculosis* against isoniazid and streptomycin while the results of rifampicin susceptibility should be complemented with DNA sequencing-based method when the suspicion for resistance is high. The possibility of false susceptibility to ethambutol with MGIT 960 system suggests that molecular or other phenotypic methods may be more useful when accurate ethambutol susceptibility results are warranted.

## Introduction

Despite declining trends in the incidence of tuberculosis (TB) in recent years, the morbidity and mortality associated with TB is still enormous and drug-resistant TB is a growing problem worldwide. According to annual surveys conducted by World Health Organization (WHO), 9.6 million new active TB cases and 1.5 million deaths occurred in 2014 [1]. Worldwide, 3.3% of new cases and 20% of previously treated cases are now identified as multidrug-resistant TB (MDR-TB, infection with *Mycobacterium tuberculosis* strain resistant at least to rifampicin and isoniazid, the two most effective first-line anti-TB drugs) [1–3]. MDR-TB is much more difficult to treat due to lengthy (18–24 months), more expensive and more toxic treatment regimens which are associated with higher rates of clinical failure and disease relapse [3–5]. MDR-TB is also a risk factor for extensively drug-resistant TB (XDR-TB, defined as infection with MDR-TB strains additionally resistant to a fluoroquinolone and injectable agent such as kanamycin, amikacin or capreomycin), a virtually untreatable disease in most of the developing countries [5–7]. Compared to the global average, much higher rates of MDR-TB (15.7% among new and 45.3% among previously treated TB cases) and XDR-TB (11.4% of all MDR-TB cases) have been reported from several countries in the European Region presenting new challenges for tuberculosis control [8–10]. Rapid and accurate laboratory diagnosis of MDR-TB is crucial for effective treatment which will also limit transmission of MDR-TB and development of XDR-TB [3, 6, 11].

Phenotypic drug susceptibility testing (DST) of *M*. *tuberculosis* is considered as the gold standard. The solid (Lowenstein-Jensen) medium-based proportion method is a WHO-recommended method but it requires 4–6 weeks to report results [12]. Commercial liquid culture systems and molecular assays have been developed and endorsed by WHO and Centers for Disease Control and Prevention (CDC) for more rapid detection of drug resistance in *M*. *tuberculosis* [11, 13–15]. The liquid-broth-based semiautomated, radiometric BACTEC 460TB (460TB) accurately performed DST of *M*. *tuberculosis* for nearly two decades, reported results within 14 days and was considered as a reliable alternative to the solid medium-based method [16–19]. The fully automated systems such as Bactec Mycobacterium Growth Indicator Tube 960 (MGIT) system, MB/BacT system and Versa TREK system with similar turnaround time subsequently replaced 460TB due to concerns for safe disposal of radioactivity [20]. Although highly consistent results were obtained between 460TB versus MGIT or other automated systems for first-line and second-line drugs during early proficiency testing studies [17–20], recent studies have shown highly discordant results for *M*. *tuberculosis* isolates carrying specific resistance conferring mutations for some first-line drugs [21–27]. However, none of these latter studies were carried out in a country from the Middle East. Since the occurrence of specific resistance conferring mutations in target genes for anti-TB drugs varies considerably across various geographical locations [6], different concordance levels may be obtained in different settings.

To further reduce the turn-around time for DST required by broth-based methods, the WHO also endorsed genotypic assays including INNO-LiPA Rif. TB and GenoType MTBDR*plus* (gMTBDR^+^) line probe assays and real-time PCR-based automated GeneXpert MTB/RIF (Xpert) assay for rapid diagnosis of TB and MDR-TB directly in clinical specimens as well as in culture isolates in developing and high-burden countries [11, 14, 15, 28]. While phenotypic assays can provide data on all first-line and second-line drugs, INNO-LiPA Rif. TB and Xpert detect resistance to rifampicin only whereas gMTBDR^+^ detects resistance to both, rifampicin and isoniazid. Resistance to rifampicin is a key determinant in treatment failure and it also generally correlates well with MDR-TB as ~85% rifampicin-resistant clinical *M*. *tuberculosis* isolates worldwide are also additionally resistant to isoniazid, [6, 11]. However, detection of resistance to rifampicin alone as a marker of MDR-TB can be problematic in some geographical locations where rifampicin monoresistance can be as high as 12% [29–31]. Furthermore, most rapid molecular tests fail to detect all clinically relevant drug resistance determinants due to the occurrence of DNA mutations outside region targeted by these tests and also yield, albeit rarely, false-positive results due to presence of silent mutations which do not affect drug efficacy [23, 24, 26, 32–36]. More recently, a genomic sequence-based scanning of drug resistance-associated loci most commonly implicated in conferring resistance to anti-TB drugs and whole genome sequencing have been performed for unambiguous and rapid determination of drug resistance of *M*. *tuberculosis* in clinical specimens and culture isolates [34, 37–42]. Although whole genome sequencing approaches could identify all drug resistance in *M*. *tuberculosis*, data complexity requiring specialist expertise has restricted their clinical application. Thus, targeted screening of a limited number of gene loci is more practical for proper management of patients with drug-resistant TB in resource-limited settings [34, 37, 42]. However, this approach requires prior knowledge of resistance conferring mutations in target genes in various settings since the prevalence of specific mutations conferring resistance to anti-TB drugs varies considerably at different geographical locations including Kuwait [6, 43–45].

This retrospective study was performed to evaluate the concordance of susceptibility results for clinical *M*. *tuberculosis* isolates across two phenotypic and two molecular methods: 460TB, MGIT, gMTBDR^+^ and targeted DNA sequencing of gene segments most commonly implicated in conferring resistance to anti-TB drugs in Kuwait, a low TB incidence country in the Middle East.

## Materials and Methods

### Patients, specimens and *M*. *tuberculosis* isolates

A total of 70 *M*. *tuberculosis* isolates grown from 70 clinical specimens (sputum, n = 49; bronchoalveolar lavage, n = 6; pus and fine needle aspirate, n = 7; tissue biopsy, n = 3; lymph node and endotracheal secretion, n = 3 and cerebrospinal fluid, n = 2) collected from 48 suspected TB patients at Kuwait National TB Reference Laboratory (KNTRL) were used. The *M*. *tuberculosis* isolates were cultured during 2006 to 2010 and were selected to include mainly MDR-TB strains from culture collection at KNTRL. The KNTRL participates in periodic drug susceptibility proficiency testing. Only two of the TB patients were Kuwaiti nationals while the remaining 46 patients were migrant workers or their family members. The clinical specimens were collected from suspected TB patients who had come to the TB clinic for treatment after obtaining verbal consent (the patient’s consent was not recorded as written consent is not required for collecting samples from patients visiting TB clinic for routine diagnosis and treatment) as part of routine diagnostic work-up and resistance surveillance. Data analyses were carried out on deidentified results. The study and the consent procedure (verbal consent for collecting clinical specimens from suspected TB patients as part of routine patient care) were approved by the Health Sciences Center Ethics Committee, Faculty of Medicine, Kuwait University (vide approval no. VDR/EC/2 dated 9-2-2015).

The smears for direct microscopy were prepared by Ziehl-Neelsen stain to detect acid-fast bacilli (AFB). Non-sterile samples were processed by using *N*-acetyl-L-cysteine and sodium hydroxide (NALC/NaOH) while sterile clinical specimens were directly processed [46]. All specimens were used for culture on solid (Lowenstein-Jensen) and MGIT system media according to the instructions supplied by the manufacturer of MGIT system (Becton Dickinson, Sparks, MD, USA) and as described previously [33, 46]. All 70 samples yielded a positive growth reading in MGIT system and the cultures were positive for the presence of AFB and for *M*. *tuberculosis* complex DNA by AccuProbe DNA probe assay which was performed as described previously [33, 46]. All 70 *M*. *tuberculosis* isolates were subjected to DST against first-line drugs by 460TB, MGIT, targeted DNA sequencing of gene segments most commonly implicated in conferring resistance to first-line drugs and for detection of MDR-TB strains by gMTBDR^+^ line probe assay. Direct microscopy, culture in MGIT system and phenotypic DST were performed at KNTRL and the personnel performing these tests were not informed of genotypic susceptibility test results. Genotypic testing was performed at the Department of Microbiology, Faculty of Medicine, Kuwait University and the personnel performing these tests were blinded to phenotypic DST results.

### Drug susceptibility testing by BACTEC 460TB system

The DST by the radiometric 460TB system was used as the gold standard and was performed in accordance with the manufacturer’s recommendations [16, 19]. The semiautomated BACTEC 460TB system was recently discontinued by the manufacturer (Becton Dickinson) due to concerns for safe disposal of radioactive substances and was replaced by the fully automated non-radiometric Bactec MGIT 960 system. The primary culture (100 μl) from MGIT tube was subcultured in a BACTEC 12B (12B) vial for susceptibility testing. The lyophilized drugs (SIRE, Becton Dickinson, Sparks, MD, USA) were reconstituted and 100 μl of each antibiotic solution was added to the labeled 12B vial. The DST assays were performed at the following final drug concentrations: 0.1 mg/L for isoniazid, 2 mg/L for rifampicin, 2.0 mg/L for streptomycin and 2.5 mg/L for ethambutol. A 12B vial broth (growth index of 500 to 800) was used for the direct inoculation of SIRE drug-containing 12B vials. A 1:100 dilution of 12B broth was also used for a SIRE drug-free control [16, 19]. All 12B vials were incubated at 37°C, tested daily in the 460TB instrument and the growth index readings were evaluated according to the established criteria for calculating susceptible, resistant and borderline DST results [16, 19].

### Drug susceptibility testing by Bactec MGIT 960 system

Susceptibility testing with the automated MGIT system was performed with MGIT cultures that tested positive at least 1 day but no more than 2 days earlier by following the manufacturer’s instructions using the SIRE drug kit [19, 47]. The lyophilized antibiotics were reconstituted in distilled water and added to MGIT tubes supplemented with 0.8 ml of the enrichment solution (Bactec MGIT SIRE supplement; Becton Dickinson). The DST assays were performed with the following final drug concentrations: 0.1 mg/L for isoniazid, 1.0 mg/L for rifampicin, 1.0 mg/L for streptomycin and 5.0 mg/L for ethambutol. All of the drug-containing tubes were inoculated with 0.5 ml of MGIT culture. A SIRE drug-free control was also inoculated with 0.5 ml of a 1:100 dilution of the positive culture broth in sterile saline. The tubes were placed in the MGIT rack, incubated in the cabinet drawer of the MGIT system and were continuously monitored. The results indicating susceptibility or resistance were interpreted and reported automatically by the MGIT system using predefined algorithms that compare bacterial growth in the drug-containing tube with the growth in the drug-free control tube [19, 47]. Repeat DST was performed on six selected isolates yielding discrepant results by the two phenotypic methods.

### Genotypic drug susceptibility testing by DNA sequencing

Genomic sequence-based scanning for drug resistance-associated mutations was performed by PCR sequencing of various segments of 6 gene loci most commonly implicated in conferring resistance to the four first-line drugs [6, 34, 37, 42]. Chromosomal DNA was extracted from MGIT culture tubes using the Chelex-100 as described previously [33] and analyzed by PCR amplification and DNA sequencing of amplicons for the presence of mutations in the 6 genes linked to resistance to rifampicin (N-terminal, cluster II and rifampicin resistance determining region or RRDR covering codons 508–534, *Escherichia coli* numbering system, of *rpoB*) [6, 32, 43, 45], isoniazid (*katG* codon 315 region and *inhA* regulatory region) [6, 43, 45], streptomycin (*rpsL* and 500 and 900 regions of *rrs*) [6, 43, 45] and ethambutol (*embB* codon 306, 406 and 497 regions) [6, 43, 45]. All 70 *M*. *tuberculosis* isolates were first tested by an in-house multiplex PCR assay specific for *M*. *tuberculosis* complex [48]. The details of various primers used for PCR amplification of various gene loci and sequencing of amplified fragments for drug resistance-associated mutations are presented in S1 Table [33, 49–51]. Amplification was performed by touchdown PCR using the reaction and cycling conditions as described previously [52]. Briefly, the PCR reaction in a final volume of 50 μl contained of 1x AmpliTaq DNA polymerase buffer, 1 unit of AmpliTaq DNA polymerase (Applied Biosystems), 0.1 mM dNTPs, 10 pmol of each primer and 2 μl of chromosomal DNA. PCR cycling conditions were same as described previously [52]. PCR products were analyzed on 2% agarose-Tris-borate-EDTA gels and stained with ethidium bromide as described previously [52]. Unincorporated primers and nucleotides were removed from PCR amplicons by using QIAquick PCR product purification kit (Qiagen, Hilden, Germany) used according to instructions supplied by the manufacturer.

Purified PCR products were sequenced with an internal forward and reverse primer at each locus for maximum coverage and reproducibility of results. The sequencing reactions were performed with an ABI BigDye terminator (version 3.1) cycle sequencing kit, as described in detail previously [33, 49]. Briefly, the reaction mixtures in a final volume of 10 μl contained 2 μl purified amplicon, 1x reaction buffer, 2 μl of BigDye terminator (version 3.1) reagent and 3.2 pmol of sequencing primer (S1 Table). The cycling parameters for sequencing reactions included an initial denaturation step at 96°C for 1 min followed by 30 cycles of 1 min at 96°C and 4 min at 60°C. The unincorporated terminators were removed from the completed sequencing reactions by using BigDye Xterminator kit (Applied Biosystems Inc.) and the samples were then loaded on an ABI 3130xl genetic analyzer for electrophoresis and data collection according to the manufacturer’s instructions.

Sequence data generated by the ABI 3130xl genetic analyzer were checked for confidence levels with an ABI sequence scanner, reverse compliments were generated and aligned with forward sequences using Clustal Omega (http://www.ebi.ac.uk/Tools/msa/clustalo/). Nucleotide and amino acid sequences were compared with the corresponding sequences from susceptible strain *M*. *tuberculosis* H_37_Rv using the Basic Local Alignment Search Tool (BLAST, National Institutes of Health, Bethesda, MD, USA; http://blast.ncbi.nlm.nih.gov/Blast.cgi?PROGRAM=blastn&PAGE_TYPE=BlastSearch&LINK_LOC=blasthome). Repeat PCR sequencing was performed on all isolates for which DNA sequencing predicted resistance but one or both of the phenotypic tests scored them as drug susceptible.

### Genotypic drug susceptibility testing by GenoType MTBDR*plus* assay

All 70 *M*. *tuberculosis* isolates were also tested by reverse hybridization-based gMTBDR^+^ assay (Hain Lifesciences, Nehren, Germany) which detects resistance to rifampicin and isoniazid only [11, 33, 53]. However, since resistance to these two drugs is sufficient for the diagnosis of MDR-TB, rapid and accurate detection of resistance to rifampicin and isoniazid is of paramount importance. Negative controls (water instead of DNA) were included with each run. The assay was performed and the results were interpreted according to the manufacturer’s recommendations and as described in detail previously [11, 33].

### Statistical analyses

Categorical variables were expressed as absolute number. Statistical analysis was performed using chi-square test or Fisher’s exact test as appropriate and probability levels <0.05 by the two-tailed test were considered as statistically significant. The strength of agreement between the different test results was assessed by using the robust kappa statistics. A kappa coefficient (κ) value of <0.4, 0.4–0.6, 0.61–0.8 and 0.81–1.0 indicated low agreement, moderate agreement, substantial agreement and perfect agreement, respectively. Statistical analyses were performed using WinPepi software ver. 3.8 (PEPI for Windows, Microsoft Inc., Redmond, WA, USA) or GraphPad software (GraphPad, La Jolla, CA, USA).

## Results

### Characteristics of *M*. *tuberculosis* isolates

We tested 70 *M*. *tuberculosis* isolates obtained from 55 pulmonary and 15 extrapulmonary specimens of 48 TB patients. One isolate each was tested from 29 patients, two isolates each were obtained from 16 patients and three isolates each were from three patients. Duplicate isolates from 19 patients were cultured within one week of isolation of the first isolate while triplicate isolates from three patients were cultured within one month of isolation of the first isolate. Repeat isolates were cultured from similar clinical specimens from 18 patients while two isolates were cultured from BAL and sputum specimens from one patient. Repeat isolates were tested to ascertain the reproducibility of the susceptibility testing. All isolates were identified as *M*. *tuberculosis* complex by the AccuProbe DNA probe assay as well as by the multiplex PCR assay based on specific amplification of two DNA fragments of ~473 bp and ~235 bp, as described previously [46, 48]. Based on DST by the 460TB, 63, 59, 34 and 43 *M*. *tuberculosis* isolates were resistant to isoniazid, rifampicin, streptomycin and ethambutol, respectively. A total of 59 (84%) isolates were multidrug-resistant and 29 (41%) isolates were resistant to all four drugs. Four isolates were polydrug-resistant while seven isolates were susceptible to all first-line drugs by 460TB.

### Discordance between genotypic drug resistance testing and phenotypic DST

The cross-tabulation of results to determine concordance between all the four methods (both phenotypic methods and both genotypic methods) for isoniazid and rifampicin and three methods (both phenotypic methods and molecular testing by DNA sequencing only since gMTBDR^+^ detects resistance to rifampicin and isoniazid only) for streptomycin and ethambutol are shown in Table 1. The agreement among all four methods for susceptibility to rifampicin and isoniazid was 96% (three discordant results) and 97% (two discordant results) of isolates, respectively. Concordance was also observed in 93% of isolates (five discordant results) for streptomycin. Only 76% of isolates (17 discordant results) yielded concordant results for ethambutol by both phenotypic methods and genotypic testing by DNA sequencing and this difference was statistically significant (*P* <0.05 for ethambutol versus rifampicin or isoniazid or streptomycin).

**Table 1:** Comparison of drug susceptibility results as determined by both phenotypic (BACTEC 460TB and MGIT 960 system) and one or both genotypic (DNA sequencing and GenoType MTBDR*plus* assay) methods.

Drug	No. of	Number of isolates scored by all methods as		
	isolates tested	Susceptible	Resistant	Discordant
Rifampicin	70	10	57	3
Isoniazid	70	7	61	2
Streptomycin	70	36	29	5
Ethambutol	70	25	28	17

In order to determine whether the discordant results were due to poor performance of one or both of the phenotypic methods compared to genotypic methods, the susceptibility test results of 460TB were compared with the results obtained by MGIT system and genotypic testing.

### BACTEC 460TB results in comparison with MGIT 960 system and genotypic susceptibility test results

Table 2 summarizes the results of 460TB in comparison with MGIT system and the two genotypic tests; DNA sequencing of different gene loci and the gMTBDR^+^ assay. The MGIT system compared to 460TB yielded 16 discrepant (false susceptible) results; two for rifampicin, one for streptomycin and 13 for ethambutol. DNA sequencing studies compared to 460TB gave 12 discrepant results. Nine isolates had false susceptibility results by DNA sequencing; two for isoniazid, five for streptomycin and two for ethambutol. Three isolates showed initial false resistance results by DNA sequencing studies; one for rifampicin and two for ethambutol. The gMTBDR^+^ assay compared to 460TB gave five discrepant results. Four isolates had false susceptibility results by gMTBDR^+^, two for rifampicin and two for isoniazid. One isolate showed initial false resistance result for rifampicin by gMTBDR^+^ assay.

**Table 2:** Drug susceptibility test results as determined by MGIT 960 system, DNA sequencing and GenoType MTBDR*plus* assay in comparison with BACTEC 460TB method.

MGIT 960 system or molecular method-based phenotype	BACTEC 460TB method-based phenotype for^b^							
	Rifampicin		Isoniazid		Streptomycin		Ethambutol	
	R	S	R	S	R	S	R	S
MGIT 960 system								
Resistant	57	0	63	0	33	0	30	0
Susceptible	2	11	0	7	1	36	13	27
DNA sequencing								
Resistant	59	1	61	0	29	0	41	2
Susceptible	0	10	2	7	5	36	2	25
GenoType MTBDR*plus*^a^								
Resistant	57	1	61	0				
Susceptible	2	10	2	7				

^a^This assay detects susceptibility to rifampicin and isoniazid only

^b^R, resistant; S, susceptible

### Concordance across phenotypic DST and genotypic testing methods

We also examined the concordance across all the methods by calculating kappa coefficients (κ) for each platform and drug and the results are shown in Table 3. Most isolates yielded nearly completely concordant results and exhibited κ values that were excellent (perfect agreement) for rifampicin (κ range, 0.84–0.95), isoniazid (κ range, 0.86–1.0) and streptomycin (κ range, 0.86–0.97) across all the tested methods, although some important differences were noted for rifampicin between the two phenotypic methods and the DNA sequence-based method (Table 3). However, diminished concordance was apparent for ethambutol resistance and was attributable largely to MGIT discrepancies (of 19 ethambutol discrepancies for 17 isolates, 15 were from MGIT and only two each were from 460TB and DNA sequencing; *P* <0.05 for MGIT system versus the other two methods). Most MGIT system ethambutol discrepancies were discrepantly susceptible (false susceptible) as 13 of 15 MGIT ethambutol susceptible results were resistant by 460TB and DNA sequencing. The κ values reiterated these findings as only moderate agreement (κ, 0.53) was exhibited with DNA sequencing and substantial agreement (κ, 0.64) with 460TB. On the contrary, perfect agreement (κ, 0.88) was exhibited by 460TB with DNA sequencing (Table 3).

**Table 3:** Kappa coefficient (κ) values across phenotypic and molecular methods.

Drug	Method	Kappa coefficient (κ) for comparison with^b^		
		BACTEC 460TB	MGIT 960 system	DNA sequencing
Rifampicin	MGIT 960 system	0.90 (0.76–1.0)		
	DNA sequencing	0.94 (0.84–1.0)	0.84 (0.67–1.0)	
	GenoType MTBDR*plus*^a^	0.84 (0.67–1.0)	0.95 (0.86–1.0)	0.89 (0.75–1.0)
Isoniazid	MGIT 960 system	1.0 (1.0–1.0)		
	DNA sequencing	0.86 (0.67–1.0)	0.86 (0.67–1.0)	
	GenoType MTBDR*plus*^a^	0.86 (0.67–1.0)	0.86 (0.67–1.0)	1.0 (1.0–1.0)
Streptomycin	MGIT 960 system	0.97 (0.92–1.0)		
	DNA sequencing	0.86 (0.74–0.98)	0.89 (0.78–0.99)	
Ethambutol	MGIT 960 system	0.64 (0.48–0.81)		
	DNA sequencing	0.88 (0.77–0.99)	0.53 (0.35–0.71)	

^a^This assay detects susceptibility to rifampicin and isoniazid only

^b^The 95% confidence interval values are also shown in parenthesis

### Summary of discordant results between phenotypic and genotypic testing methods

A total of 24 *M*. *tuberculosis* isolates exhibited a discordant result between phenotypic and genotypic methods. The resolution of discrepant results was accomplished by comparison of the drug susceptibility testing data by all the four methods and the results are presented in Table 4. Of the three *M*. *tuberculosis* isolates yielding discrepant results for rifampicin, two (5177/06 and 9049/06) were resistant by 460TB and DNA sequencing but susceptible by MGIT and gMTBDR^+^ assay. These isolates contained I572F mutation in cluster II region of the *rpoB* gene. One isolate was susceptible by both phenotypic methods but was resistant by both gMTBDR^+^ and DNA sequencing. This isolate (13242/10) contained D516Y mutation in *rpoB* gene. Two isolates (1171/08 and 3130/08) were resistant to isoniazid by both phenotypic methods but isoniazid susceptible by both genotypic methods. Five isolates (2496/07, 2609/07, 3805/07, 1572/09, and 8132/10) yielded discrepant results for streptomycin and four of these five isolates were resistant to streptomycin by both phenotypic methods but susceptible by DNA sequencing. One isolate (1572/09) was resistant by 460TB but susceptible by both MGIT and DNA sequencing (Table 4). A total of 17 isolates yielded discrepant results for ethambutol and 13 of these 17 isolates were resistant by 460TB and DNA sequencing but susceptible by MGIT (Table 4). Two isolates (193/09 and 676/09) were susceptible by both phenotypic methods but ethambutol-resistant by DNA sequencing while two other isolates (4519/06 and 4556/06) were resistant by both phenotypic methods but susceptible by DNA sequencing.

**Table 4:** Summary of discrepant results for 24 *M*. *tuberculosis* isolates from BACTEC 460TB and MGIT 960 system in comparison with the results from GenoType MTBDR*plus* assay and DNA sequence-based method.

Serial	Isolate	Resistance patterns obtained during drug susceptibility testing with^a^				Mutations detected by DNA sequencing in						Final
no.	no.	460TB	MGIT	DNA sequencing	gMTBDR^+^^b^	*rpoB*	*katG*	*inhA*	*rpsL*	*rrs*	*embB*	resistance
1	3070/06	SIRE	SIR	SIRE	IR	D516V	S315T	None	K43R	None	Q497K	SIRE
2	3122/06	SIRE	SIR	SIRE	IR	D516V	S315T	None	K43R	None	Q497K	SIRE
3	4519/06	IRE	IRE	IR	IR	Q513L	S315N	None	None	None	None	IRE
4	4556/06	IRE	IRE	IR	IR	Q513L	S315N	None	None	None	None	IRE
5	5177/06	IRE	IE	IRE	I	I572F	S315T	None	None	None	M306I	IRE
6	9049/06	IRE	IE	IRE	I	I572F	S315T	None	None	None	M306I	IRE
7	688/07	IRE	IR	IRE	IR	S531L	S315T	-8T/A	None	None	M306I	IRE
8	789/07	IRE	IR	IRE	IR	S531L	S315T	-8T/A	None	None	M306I	IRE
9	2496/07	SIRE	SIR	IRE	IR	M515I + D516Y	S315T	None	None	None	G406C	SIRE
10	2609/07	SIRE	SIR	IRE	IR	M515I + D516Y	S315T	None	None	None	G406C	SIRE
11	2622/07	IRE	IR	IRE	IR	M515I + D516Y	S315T	None	None	None	G406C	IRE
12	4613/07	IRE	IR	IRE	IR	S531L	S315T	None	None	None	M306I	IRE
13	4753/07	IRE	IR	IRE	IR	S531L	S315T	None	None	None	M306I	IRE
14	3805/07	SIRE	SIRE	IRE	IR	S531L	S315T	None	None	None	Q497R	SIRE
15	8000/07	IRE	IR	IRE	IR	H526D	None	-15C/T	None	None	M306L	IRE
16	1171/08	IR	IR	R	R	S531L	None	None	None	None	None	IR
17	3130/08	SIRE	SIRE	SRE	R	S531L	None	None	None	G878A	Q497K	SIRE
18	193/09	IR	IR	IRE	IR	H526D	S315T	None	None	None	M306I	IRE
19	676/09	IR	IR	IRE	IR	H526D	S315T	None	None	None	M306I	IRE
20	1572/09	SIRE	IR	IRE	IR	H526D	S315T	None	None	None	M306I	SIRE
21	5636/10	SIRE	SIR	SIRE	IR	S531L	S315T	None	K43R	None	G406S	SIRE
22	7596/10	SIRE	SIR	SIRE	IR	S531L	S315T	None	K43R	None	G406S	SIRE
23	8132/10	SIR	SIR	IR	IR	S531L	S315T	None	None	None	None	SIR
24	13242/10	IE	IE	IRE	IR	D516Y	S315T	None	None	None	M306V	IRE

^a^460TB, BACTEC 460TB; MGIT, MGIT 960 system; gMTBDR^+^, GenoType MTBDR*plus* assay; S, streptomycin; I, isoniazid; R, rifampicin; E, ethambutol

^b^This assay detects susceptibility to rifampicin and isoniazid only

### Reproducibility testing

Duplicate (and triplicate) isolates from the same patient yielded identical results as the first isolate by both phenotypic and genotypic tests. Repeat phenotypic tests performed on six selected isolates yielding discrepant results exhibited the same pattern of susceptibility as that obtained on first testing. Furthermore, repeat PCR sequencing performed on all isolates for which initial DNA sequencing predicted resistance but one or both of the phenotypic tests scored them as drug susceptible yielded the same mutation in the target gene.

## Discussion

Kuwait, an Arabian Gulf country in the Middle East (TB incidence of 24 cases per 100,000 population) is a low TB incidence country [46]. Culture on solid medium and automated (MGIT) liquid culture system are routinely used for definitive diagnosis of active TB disease. Phenotypic DST is performed on all *M*. *tuberculosis* isolates and ~1.5% of the isolates in Kuwait are detected as MDR-TB strains [36, 46]. Since 2011, all clinical specimens from suspected TB patients are tested by Xpert assay in addition to routine processing for smear microscopy and culture. Furthermore, MGIT completely replaced the 460TB in 2011 for DST of all *M*. *tuberculosis* isolates. However, the reliability of DST by MGIT has not been evaluated in Kuwait. Rapid detection of resistance of *M*. *tuberculosis* isolates to isoniazid and rifampicin is crucial for timely diagnosis and management of MDR-TB [6, 11, 14, 15, 34]. Hence, there is increasing emphasis on determination of drug resistance through molecular methods as they can provide results within days versus weeks required for phenotypic DST [6, 11, 15, 34, 41, 42]. In this study we evaluated the performance of two phenotypic (460TB and MGIT systems) and two molecular (gMTBDR^+^ and DNA sequencing) methods for accurately detecting resistance of *M*. *tuberculosis* isolates in Kuwait and we found discordance for all four first-line anti-TB drugs.

In our study, we initially used DST results by 460TB as the ‘gold standard’ as this method has detected resistance of *M*. *tuberculosis* isolates to all four first-line drugs accurately throughout the world including Kuwait for nearly two decades [16–20]. A total of 70 *M*. *tuberculosis* isolates obtained from both pulmonary and extrapulmonary specimens obtained from 48 TB patients were tested. Repeat isolates were tested to ascertain the reproducibility of results obtained with the four methods. Most (63 of 70) were drug-resistant isolates including 59 (84%) MDR-TB strains by 460TB. Concordance among all four methods for susceptibility to rifampicin, isoniazid and streptomycin was 96%, 97% and 93%, respectively, while significantly less (76%) isolates yielded concordant results for ethambutol. Although accurate detection of resistance to rifampicin and isoniazid is most crucial for rapid detection of MDR-TB strains, susceptibility data for streptomycin and ethambutol are also important when formulating individualized treatment regimens for patients with MDR-TB since inclusion of as many active first-line drugs as possible minimizes the number of toxic and less-effective second-line drugs in treatment regimens [4–6, 54]. The discordant results for each anti-TB drug can be either truly resistant, with the gold standard method indicating susceptibility (false susceptible) or actually susceptible, with the gold standard method indicating resistance (false resistance). For the resolution of discrepant results in the final analysis, we used the presence of well-known resistance-conferring mutations in target genes as indicative of resistance even when 460TB or both phenotypic methods indicated susceptibility (Table 4). When the resolution of discrepant results was not possible, DST data by 460TB was retained (final result; [17–20]) for isolates lacking a mutation in the target genes since only a few loci were analyzed in this study whereas multiple loci are implicated in conferring resistance to all four first-line drugs [38–45].

For rifampicin, 67 isolates showed concordant results by all four methods while three isolates yielded discordant results. Two of these isolates that were rifampicin-resistant by 460TB and DNA sequencing showed the presence of I572F mutation in *rpoB* gene. Both isolates were rifampicin-susceptible by MGIT and MTBDR^+^, however, the latter assay is not designed for the detection of this mutation as it is outside RRDR, the target region for gMTBDR^+^ assay [11, 15, 53]. Another isolate was rifampicin-susceptible by both phenotypic methods but was rifampicin-resistant by DNA sequencing and gMTBDR^+^ assay with sequencing data showing the presence of D516Y mutation in RRDR of the *rpoB* gene. More importantly, this isolate (13242/10) was detected as MDR-TB strain by both molecular methods. Interestingly, both D516Y and I572F, similar to few other ‘disputed’ mutations (like L511P, H526D, H526L, H526Y, H526N, L533P) in the *rpoB*, confer low-level but clinically significant resistance to rifampicin which are often missed by growth-based methods, particularly the automated liquid culture systems [21, 23, 24, 26, 27]. Thus, 3 (4%) of 70 *M*. *tuberculosis* isolates in Kuwait contained *rpoB* mutations that confer low-level resistance to rifampicin. However, the exact prevalence of these mutations among all *M*. *tuberculosis* isolates in Kuwait remains undetermined since the selected isolates mainly included MDR-TB strains. Treatment of patients infected with these low-level rifampicin-resistant *M*. *tuberculosis* isolates is challenging as they are detected as rifampicin-susceptible by the conventional phenotypic tests yet the patients often relapse or fail treatment [24, 55, 56]. It has recently been suggested that both phenotypic and molecular test results should be considered for the diagnosis of MDR-TB [23–25]. Our results are in agreement with these observations as three of our 60 MDR-TB isolates were only detected as polydrug-resistant by one or both phenotypic methods. The results also highlight the incapability of MGIT system in detecting these low-level rifampicin-resistant strains. Although the occurrence of these ‘disputed’ mutations among clinical *M*. *tuberculosis* isolates remains unknown, it could be considerable, particularly among patients with clinical suspicion of drug resistance as was recently shown in one study involving TB patients from Bangladesh and Kinshasa [24]. The ‘disputed’ mutations accounted for >10% of all *rpoB* mutations in *M*. *tuberculosis* strains from patients with failing therapy or experiencing relapse and the frequency of treatment failure or relapse was same in patients infected with strains with well-characterized or ‘disputed’ *rpoB* mutations [24]. Consistent with other recent reports, our data also suggest adaptation of the standard phenotypic DST by MGIT for greater accuracy of rifampicin resistance detection [23, 24, 26, 27] and a susceptible result should be confirmed by molecular testing when the suspicion for rifampicin resistance (such as previous history of anti-TB therapy, failing therapy, relapse or history of close contact with a patient with rifampicin-resistant/MDR-TB) is very high.

All 70 isolates yielded completely concordant results for isoniazid between the two genotypic methods. This is not unexpected since DNA sequencing was carried out only for *katG* gene region around codon 315 and *inhA* regulatory region which are also the targets (*katG* codon 315 and *inhA* regulatory region) of gMDBDR^+^ assay [11, 15, 33, 53]. Also, all isolates yielded completely concordant results between the two (460TB and MGIT) phenotypic methods which is in line with previous studies reporting nearly concordant results for isoniazid susceptibility by these two methods [17–19, 57]. However, 68 isolates yielded concordant and two isolates yielded discordant results between the phenotypic and genotypic methods with genotypic methods scoring both the isolates as susceptible. This result is also expected since mutations in other regions of *katG* and *inhA* genes as well as mutations in few other genes occur in 2–10% of all isoniazid-resistant *M*. *tuberculosis* isolates [34, 42–45, 58, 59].

Although streptomycin is now used as a second-line drug due to availability of other first-line drugs that can be conveniently used in combination therapy, it is bactericidal for *M*. *tuberculosis* with fewer side-effects than other second-line drugs and is a desirable drug to include in multi-drug regimens for the treatment of drug-resistant TB/MDR-TB provided that the isolate is susceptible to streptomycin [6, 54, 60]. Furthermore some XDR-TB strains have also been reported that remain susceptible to streptomycin since there is usually little cross-resistance with other injectable aminoglycosides (kanamycin and amikacin) or cyclic polypeptides (capreomycin and viomycin), making streptomycin suitable even for the treatment of some XDR-TB patients [45, 61]. The DST for streptomycin was carried out by two phenotypic methods (460TB and MGIT) but only one (DNA sequencing) genotypic method since gMDBDR^+^ assay detects resistance to rifampicin and isoniazid only. Nearly all (69 of 70, 99%) isolates yielded concordant results between the two phenotypic methods while one isolate was streptomycin-resistant by 460TB but streptomycin-susceptible by MGIT. Although DNA sequencing could not resolve the discrepant result, the isolate was considered as streptomycin-resistant in the final analysis since previous studies have shown that MGIT has a significantly increased risk of reporting false-negative result for streptomycin compared to 460TB [19, 20]. Four other isolates yielded discrepant results where both phenotypic methods showed resistance while DNA sequencing did not detect a resistance conferring mutation. This can be explained by the fact that only two (*rpsL* and *rrs*) loci were studied for mutations while resistance to streptomycin is mediated by few other (*gidB*, efflux pumps etc.) genes in *M*. *tuberculosis* [6, 42, 62, 63]. It is therefore probable that the basis of resistance in these isolates was due to an alteration in other genes which were not investigated.

False susceptibility to ethambutol is not very critical for the treatment of drug-susceptible TB since ethambutol is used only in the initiation phase of treatment and can even be omitted from the drug regimen once susceptibility of *M*. *tuberculosis* isolate to rifampicin and isoniazid has been documented [64]. However, false susceptibility to ethambutol is of considerable importance for the successful treatment of MDR-TB as treatment regimens for these patients should include any active first-line drug for improved outcome [5, 6, 54]. Similar to few other studies [20, 25, 65, 66], the maximum number of discrepant results was obtained for ethambutol, all involving MDR-TB strains. In total, 17 isolates yielded discrepant results and 13 of these 17 isolates were resistant by 460TB and DNA sequencing but susceptible by MGIT while two other isolates were susceptible by both phenotypic methods but resistant by DNA sequencing of *embB*. Ethambutol is a slow-acting anti-TB drug and susceptibility testing to ethambutol has been problematic with liquid culture-based methods [20, 67]. The MGIT often reports false ethambutol susceptibility for *M*. *tuberculosis* isolates containing *embB* mutations that confer low-level but clinically significant resistance to ethambutol [63, 68–70]. Our results support molecular testing for detecting ethambutol resistance in multidrug-resistant *M*. *tuberculosis* isolates where accurate ethambutol susceptibility results are warranted. Two isolates were ethambutol-resistant by both phenotypic methods but susceptible by DNA sequencing. Although mutations at *embB* codon 306, 406 and 497 occur most frequently among ethambutol-resistant *M*. *tuberculosis* isolates, other regions of *embB* as well as mutations in several other genes are also involved in conferring resistance to ethambutol [42, 44–46, 71]. Thus, it is likely that the molecular basis of resistance to ethambutol in these two isolates involves an alteration in other genes/gene segment that was not interrogated in this study.

## Conclusions

The data reported in this study have clearly shown that compared to BACTEC 460TB, the MGIT 960 system is an accurate alternative method for DST of *M*. *tuberculosis* against isoniazid and streptomycin. However, the liquid culture-based methods, particularly MGIT 960 system, fail to detect low-level yet clinically significant rifampicin resistance. Thus, molecular testing (such as DNA sequencing of *rpoB*) for rifampicin resistance detection should be employed for all polydrug-resistant strains or when the suspicion for resistance (such as failing therapy or relapse) is high. The increased possibility of false susceptibility to ethambutol with MGIT 960 system suggests that molecular methods may be more useful when accurate ethambutol susceptibility results are warranted.

## Supporting Information

S1 Table Nucleotide sequence of primers used to amplify and sequence various specific regions of *M*. *tuberculosis* DNA for detection of drug resistance conferring mutations in target genes. (DOCX)
