# Supplementary material for: Discordance across Phenotypic and Molecular Methods for Drug Susceptibility Testing of Drug-Resistant Mycobacterium tuberculosis Isolates in a Low TB Incidence Country
Source: PLoS One. 2016 Apr 20;11(4):e0153563. doi: 10.1371/journal.pone.0153563 (PMC4838278; doi:10.1371/journal.pone.0153563)
Supplement: S1 Table — (DOCX) [file pone.0153563.s001.docx]

**S1 Table.** Nucleotide sequence of primers used to amplify and sequence various specific regions of *M. tuberculosis* DNA for detection of drug resistance conferring mutations in target genes.

| Primer | Target | Target | Direction | Oligonucleotide sequence | Purpose | Product | Reference |
| --- | --- | --- | --- | --- | --- | --- | --- |
|  | gene^a^ | region^b^ |  |  |  | size^c^ (bp) |  |
| RPOHSF | *rpoB* | RRDR | Forward | 5’- GACGACATCGACCACTTCGGCAAC -3’ | PCR |  | 49 |
| RPOHSR | *rpoB* | RRDR | Reverse | 5’- GAACGGGTTGACCCGCGCGTACA -3’ | PCR | 426 | 49 |
| RPOHSFS | *rpoB* | RRDR | Forward | 5’- AAACCAGATCCGGGTCGGCATGT -3’ | Sequencing | N. A. | 49 |
| RPOHSRS | *rpoB* | RRDR | Reverse | 5’- GCGTACACCGACAGCGAGCCGA -3’ | Sequencing | N. A. | 49 |
| RPONF | *rpoB* | N-terminal | Forward | 5'- CGACGAGTGCAAAGACAAGGACA -3' | PCR |  | 49 |
| RPONR | *rpoB* | N-terminal | Reverse | 5'- GACGGTGTCGCGCTTGTCGAC -3' | PCR | 310 | 49 |
| RPONFS | *rpoB* | N-terminal | Forward | 5'- TTCGTCACCGCCGAGTTCATCAA -3' | Sequencing | N. A. | 49 |
| RPONRS | *rpoB* | N-terminal | Reverse | 5'- CTTGACGCTGTGCAGCGTCTTGT -3' | Sequencing | N. A. | 49 |
| RPOIIF | *rpoB* | Cluster II | Forward | 5'- TCATGGACCAGAACAACCCGCTGT -3' | PCR |  | 33 |
| RPOIIR | *rpoB* | Cluster II | Reverse | 5'- CGTTGTCGTGCATCACAGTGATGT -3' | PCR | 678 | 33 |
| RPOIIFS | *rpoB* | Cluster II | Forward | 5'- CGCGACGTGCACCCGTCGCACT -3' | Sequencing | N. A. | 33 |
| RPOIIRS | *rpoB* | Cluster II | Reverse | 5'- GGCACCGCCTGGCGCTGGATGTT -3' | Sequencing | N. A. | 33 |
| KatG315F | *katG* | Codon 315 | Forward | 5’- CCCATGGCCGCGGCGGTCGACATT -3’ | PCR |  | 50 |
| KatG315R | *katG* | Codon 315 | Reverse | 5’- CGCCGTCCTTGGCGGTGTATTGCC -3’ | PCR | 355 | 50 |
| KatG315FS | *katG* | Codon 315 | Forward | 5’- CACACTTTCGGTAAGACCCA -3’ | Sequencing | N. A. | This study |
| KatG315RS | *katG* | Codon 315 | Reverse | 5’- AGCAGGGCTCTTCGTCAGCT -3’ | Sequencing | N. A. | This study |
| INHAF | *inhA* | Regulatory region | Forward | 5’- CGACATACCTGCTGCGCAATTCGT -3’ | PCR |  | 33 |
| INHAR | *inhA* | Regulatory region | Reverse | 5’- CGTCGCTGTCGGTGACGTCACAT -3’ | PCR | 418 | 33 |
| INHAFS | *inhA* | Regulatory region | Forward | 5’- CAGAAAGGGATCCGTCATGGT -3’ | Sequencing | N. A. | 33 |
| INHAFR | *inhA* | Regulatory region | Reverse | 5’- CAGCCGCTGTGCGATCGCCA -3’ | Sequencing | N. A. | 33 |
| RPSLF | *rpsL* | Nearly entire gene | Forward | 5'- CGCAAGGGTCGTCGGGACAAGA -3' | PCR |  | This study |
| RPSLR | *rpsL* | Nearly entire gene | Reverse | 5`- GGTGACCAACTGCGATCCGTAGA- 3` | PCR | 422 | This study |
| RPSLFS | *rpsL* | Nearly entire gene | Forward | 5`- GGACAAGATCAGTAAGGTCAA- 3` | Sequencing | N. A. | This study |
| RPSLRS | *rpsL* | Nearly entire gene | Reverse | 5`- CGTTGACCAACGGACGCTTGG -3` | Sequencing | N. A. | This study |
| 16S500F | *rrs* | 500 region | Forward | 5`- GGCCTTCGGGTTGTAAACCTCTT -3` | PCR |  | This study |
| 16S500R | *rrs* | 500 region | Reverse | 5`- GCATTCCACCGCTACACCAGGAA -3` | PCR | 276 | This study |
| 16S500FS | *rrs* | 500 region | Forward | 5`- ACCTCTTTCACCATCGACGAA -3` | Sequencing | N. A. | This study |
| 16S500RS | *rrs* | 500 region | Reverse | 5`- CAGTACTCTAGTCTGCCCGTAT -3` | Sequencing | N. A. | This study |
| 16S900F | *rrs* | 900 region | Forward | 5`- GGATTAGATACCCTGGTAGTCCA -3` | PCR |  | This study |
| 16S900R | *rrs* | 900 region | Reverse | 5`- GGTTGCGCTCGTTGCGGGACTTA -3' | PCR | 329 | This study |
| 16S900FS | *rrs* | 900 region | Forward | 5`- CGCCGTAAACGGTGGGTACTA -3` | Sequencing | N. A. | This study |
| 16S900RS | *rrs* | 900 region | Reverse | 5`- GACACGAGCTGACGACAGCCAT -3` | Sequencing | N. A. | This study |
| EMB306F | *embB* | Codon 306/406 | Forward | 5’- CCGACGCCGTGGTGATATTCGGCT- 3’ | PCR |  | 51 |
| EMB406R | *embB* | Codon 306/406 | Reverse | 5’- CAGTGTGAATGCGGCGGTAACGAC -3’ | PCR | 492 | This study |
| EMB306FS | *embB* | Codon 306 | Forward | 5’- CCGTGGTGATATTCGGCTT -3’ | Sequencing | N. A. | This study |
| EMB306RS | *embB* | Codon 306 | Reverse | 5’- AGCAGCCAGCACACTAGCC -3’ | Sequencing | N. A. | This study |
| EMB406FS | *embB* | Codon 406 | Forward | 5’- TGCTGGCTGCTGCTGTCGCGTGA -3’ | Sequencing | N. A. | This study |
| EMB406RS | *embB* | Codon 406 | Reverse | 5’- TGTGAATGCGGCGGTAACGACGG -3’ | Sequencing | N. A. | This study |
| EMB497F | *embB* | Codon 497 | Forward | 5’- GGATCTTGGTGCGCCGTCATCG -3’ | PCR |  | This study |
| EMB497R | *embB* | Codon 497 | Reverse | 5’- GTGAACAGGCATAGCGCGGTGA -3’ | PCR | 265 | This study |
| EMB497FS | *embB* | Codon 497 | Forward | 5’- GTGCGCCGTCATCGCCTGGT -3’ | Sequencing | N. A. | This study |
| EMB497RS | *embB* | Codon 497 | Reverse | 5’- AACCGTCGACGGTGGGCAGGAT -3’ | Sequencing | N. A. | This study |

^a^GenBank/EMBL/DDBJ accession numbers for the target genes: *rpoB*, L27989; *katG*, X68081; *inhA*, U66801; *rpsL*, X70995; *rrs*, X58890 and *embB*, Z80343.

^b^RRDR, rifampicin resistance determining region (*rpoB* codons 508-534, *E. coli* numbering system [32]); N-terminal, N-terminal region of *rpoB* including codon V146; Cluster II, cluster II region of *rpoB* including codons 561-672; Regulatory region, *mabA-inhA* promoter region

^c^Product size corresponds to PCR amplified product obtained with each forward and reverse primer set used for PCR amplification
